# Molecular Dynamic Simulations Suggest That Metabolite-Induced Post-Translational Modifications Alter the Behavior of the Fibrinogen Coiled-Coil Domain

**DOI:** 10.3390/metabo11050307

**Published:** 2021-05-11

**Authors:** Zofie Sovova, Jiri Suttnar, Jan E. Dyr

**Affiliations:** Department of Biochemistry, Institute of Hematology and Blood Transfusion, U Nemocnice 1, 128 00 Prague, Czech Republic; jiri.suttnar@uhkt.cz (J.S.); jan.dyr@uhkt.cz (J.E.D.)

**Keywords:** fibrinogen, post-translational modifications, molecular dynamic simulation, oxidation, citrullination, coiled-coil

## Abstract

Fibrinogen is an abundant blood plasma protein that, inter alia, participates in blood coagulation. It polymerizes to form a fibrin clot that is among the major components of the thrombus. Fibrinogen reactions with various reactive metabolites may induce post-translational modifications (PTMs) into the protein structure that affect the architecture and properties of fibrin clots. We reviewed the previous literature to find the positions of PTMs of fibrinogen. For 7 out of 307 reported PTMs, we used molecular dynamics simulations to characterize their effect on the behavior of the fibrinogen coiled-coil domain. Interactions of the γ-coil with adjacent chains give rise to π-helices in Aα and Bβ chains of even unmodified fibrinogen. The examined PTMs suppress fluctuations of the γ-coil, which may affect the fibrinolysis and stiffness of the fibrin fibers. Citrullination of AαR104 and oxidations of γP70 and γP76 to glutamic semialdehyde unfold the α-helical structure of Aα and Bβ chains. Oxidation of γM78 to methionine sulfoxide induces the formation of an α-helix in the γ-coil region. Our findings suggest that certain PTMs alter the protein secondary structure. Thus, the altered protein structure may indicate the presence of PTMs in the molecule and consequently of certain metabolites within the system.

## 1. Introduction

Various (bio)chemical processes, including cellular respiration, cell replication and pathogen defense, are ongoing in all living organisms. Many reactive metabolites, like hydrogen peroxide (H_2_O_2_), hypochlorite (ClO^−^), allysine or homocysteine, are produced by these reactions. Some of the metabolites are radicals (i.e., atoms or molecules owning at least one unpaired electron) or have unstable bonds, which makes them reactive. Under physiological conditions, these metabolites, known as reactive species (RS), participate in cell signaling, host defense and biosynthetic processes [1]. Too high or too low a level of RS induces pathophysiological processes. An excess of RS causes damage to other molecules. This is manifested by, for example, neurological and autoimmune diseases. A scarcity of RS disturbs the processes in which they are involved, which may be demonstrated by a decrease in antimicrobial defense, or low blood pressure [1,2]. The overabundance or deficiency of RS is referred to as oxidative stress [2].

RS readily react with other molecules. Their reactions with side chains and termini of proteins give rise to post-translational modifications (PTMs) and at high concentration, some RS can cleave the protein backbone. PTMs are covalent changes in protein structure that alter either the stereochemical and/or electrostatic properties of protein and may indicate the presence of a certain metabolite in the system. They are also indicators of numerous diseases, like neurodegenerative diseases, cardiovascular diseases and cancer. Apart from RS, some PTMs are enzymatically induced into the protein structure. In blood, fibrinogen is among the most abundant targets for RS [3] and is used as a model molecule for PTM detection [4,5,6].

Oxidation, as a protein PTM, is a reaction of a protein with a reactive oxygen species (ROS). ROS, like H_2_O_2_, the superoxide anion radical (O_2_^•-^) and ozone (O_3_), are the most extensively studied groups of RS, due to their high abundance. Apart from an exogenous source, the mitochondrial electron transport chain is the major source of ROS in mammals. Oxygen can escape from the mitochondrial electron transport chain in the form of O_2_^•-^ which in a superoxide dismutase-catalyzed reaction is converted to the less reactive H_2_O_2_. Catalase catalyzes the subsequent decomposition of H_2_O_2_ to O_2_. ROS are also produced by numerous enzymes (e.g., NADPH oxidases and cytochrome P450) and can be released by neutrophils in pathogen defense (e.g., ClO^−^ is synthesized from O_2_ via H_2_O_2_ in a reaction catalyzed by myeloperoxidase, O_3_, that is generated in an antibody-catalyzed pathway [2,7,8]).

The sulfur-containing amino acids (AAs) methionine and cysteine are, due to the low oxidative potential of sulfur, the AAs most vulnerable to ROS [9]. As these two residues are the only AAs where oxidation can be enzymatically reversed [10], they have been proposed to act as antioxidants in proteins. The relatively high susceptibility of tyrosine, tryptophan, histidine, and phenylalanine to oxidation is explained by the abundance of electrons in their aromatic rings. The oxidation of lysine, arginine, proline, threonine, and asparagine, which is often catalyzed by a metal or an enzyme, is relatively rare. It is usually reported in in vitro oxidized samples, where higher doses of oxidizing agents are used. It may point to a high level of ROS in the system [10]. The oxidative modifications of fibrinogen are associated with numerous diseases, like myocardial infarction, Behçet disease, cirrhosis, and trauma-induced coagulopathy [11]. The effect of oxidation on fibrinogen behavior differs among experiments, which is explained by varying experiment settings [11,12]. In most cases, the oxidation of fibrinogen decreases its polymerization rate, clot lysis, maximum turbidity, the diameter of fibrin fibers, the stiffness of the clot, and its permeability. On a microscale level, oxidation decreases the content of α-helices in the fibrinogen structure and of CH_2_ and CH_3_ moieties, and increases the content of carbonyl groups in sidechains [13].

O_2_^•-^ reacts with nitric oxide (NO^•^) to form peroxynitrite (ONOO^−^), which is another RS participating in pathogen defense and cellular signaling [14]. In mammals, NO^•^ is a metabolite of a nitric oxide synthases-catalyzed conversion of free arginine to citrulline. Peptidyl arginine is converted to citrulline as well, although this reaction is catalyzed by peptidyl arginine deiminases (PAD) and produces ammonium as a metabolite. This PTM is abundant in patients with inflammation and autoimmune diseases, like rheumatoid arthritis [15]. Citrullination prevents fibrinopeptide cleavage, and thus decreases the rate of fibrin polymerization [16]. Fibrin clots made of a mixture of citrullinated and native fibrinogen have a lower fibrin fiber density and are made of thinner fibers [11].

Another metabolic pathway possibly resulting in a PTM is the catabolism of methionine, followed by the metabolism of homocysteine (Hcy). The methionine in the so-called methionine cycle is enzymatically converted to Hcy, which can be converted to cysteine via the transsulfuration pathway. This pathway is catalyzed by cystathionine β-synthase and cystathionase. Alternatively, Hcy can undergo a methionyl-tRNA synthase-catalyzed reaction to be converted to homocysteine thiolactone, that subsequently reacts with the amine group of lysine, forming a homocysteine-lysine adduct [17]. The latter reaction, which dominates in individuals with impaired cystathionine β-synthase, leads to the cumulation of Hcy in an organism and is manifested by hyperhomocysteinemia. *N*-homocysteinylation aids protein aggregation and makes them prone to oxidation.

**Figure 1:**
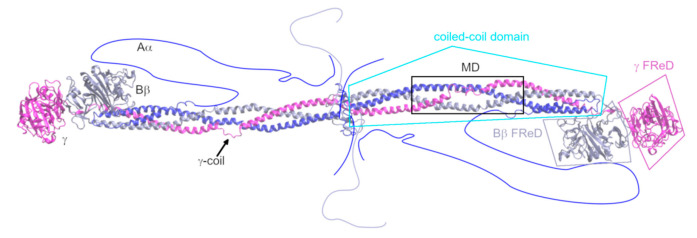
Structure of fibrinogen, based on its crystal structure 3GHG [18]. The missing regions are schematically drawn into the figure. The region that is the subject of this study is highlighted by a black square and designated as “MD”. The Aα chain is shown in blue, the Bβ chain in grey-violet, and the γ chain in magenta. The domains of fibrinogen are designated. FReD stands for “fibrinogen-related domain”.

Fibrinogen (Figure 1) is, in its major form, an abundant blood plasma glycoprotein of 340 kDa resp. 2964 AAs. It is formed by two heterotrimers composed of the chains Aα (610 AAs in mature form), Bβ (461 AAs), and γ (411 AAs), which are oriented by their N-terminals toward each other [18]. There are two prominent structural regions in the fibrinogen molecule: one triple coiled-coil domain and two fibrinogen-related domains. The parallel triple coiled-coil is formed by AAs AαG48–AαC161, BβG79–BβS187, and γT22–γA133, and is interrupted by an 11-residue coil (γY68–γM78) in the γ chain. This interruption is hereafter referred to as the γ-coil. It was shown [18,19] that the γ-coil facilitates bending of the whole coiled-coil domain, and plasmin cleavage sites were detected in the vicinity of the γ-coil [20]. The α-helices of the coiled-coil domain have long been thought to be linked by interchain disulfide bridges at their termini. Other disulfide bridges were thought to join the two trimers together. Recently, it was shown [4] that some of these disulfides may be reduced, i.e., the two binding partners do not form a covalent bond.

The coiled-coil is a structural motif that occurs in 5–10% of proteins among all domains of life [21]. A heptad *abcdedg*, where AAs at positions *a* and *d* are hydrophobic, and the others are polar, is a characteristic sequence motif for coiled-coils. Helices are oriented by their hydrophobic residues toward each other in a so-called knob-into-hole fashion, where residues at positions *a* and *d* are the knobs and residues *d_-1_*, *g_-1_*, *a*, and *d* of the other helix form a diamond-shaped hole [22]. The N-termini of fibrinogen chains are disordered, and 16 resp. 14 N-terminal AAs of the Aα resp. Bβ chains are known as fibrinopeptides A resp. B. The coiled-coil domain is in the Bβ and γ chains, followed by a fibrinogen-related domain (for its structural description, see [23]). In the Aα chain, it is succeeded by a mainly disordered region. Aα and γ chains are alternately spliced to γ’ and Fib_420_ forms. The former has an extension of 16 amino acids on its C-terminus, and 4 C-terminal amino acids of the major form are altered. This form occurs in 8–15% of molecules of fibrinogen [24]. The Fib_420_ has a 236-AA extension of the Aα chain, forming a fibrinogen-related domain, and this form occurs in 2% of fibrinogen molecules [25].

The enzymatic cleavage of fibrinopeptides turns soluble fibrinogen into insoluble fibrin. Fibrinopeptides are cleaved by serine protease thrombin under physiological conditions. Fibrin polymerizes into two-stranded elongated polymers, known as protofibers, which form thicker fibrin fibers. The resultant structure of fibrin polymerization is a fibrin clot that acts as a matrix for thrombus [26]. Thrombus, under physiological conditions, prevents bleeding; however, under pathophysiological conditions, it participates in various cardiovascular diseases, like thrombosis, stroke, and myocardial infarction. This work focuses on the impact of PTMs, namely, oxidation and citrullination, on fibrinogen structure and properties.

Recently, we have shown [27] that molecular dynamic (MD) simulations can capture the initial stages of protein structural changes induced by PTMs. This work extends the previous report by describing the impact of an additional 7 PTMs on the coiled-coil domain of fibrinogen. We are dealing with the citrullination of AαR104 (designated as AαR(Cit)104) and oxidations of γP70 to glutamic semialdehyde (γP(Ox)70), of γK75 to allysine (γK(Ox)75), of γP76 to glutamic semialdehyde (γP(Ox)76) and to pyroglutamic acid (γP(Ox)76PGA), of γM78 to methionine sulfoxide (γM(Ox)78) and with the change of γN77 to aspartic acid (γN(Ox)77). The knowledge of the impact of PTMs on the atomistic structure of fibrinogen helps in understanding and predicting the influence of PTMs of fibrin fiber properties on the affected characteristics of thrombi. This study also helps in understanding the behavior of coiled-coil domains. The assignment of certain structural changes within the protein to a defined PTM may point to the presence of a metabolite inducing the given PTM in the system.

## 2. Results

### 2.1. PTMs Reported in Fibrinogen

**Figure 2:**
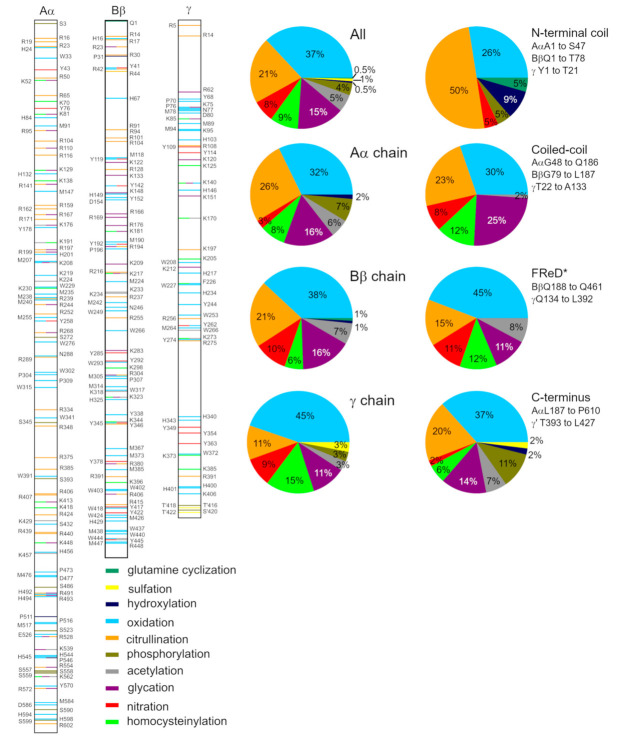
Position and character of PTMs as reported in the literature. For detailed information about experiments reporting these PTMs, see Table S1. *FReD stands for “fibrinogen-related domain”.

We found 253 positions of 307 PTMs, reported in 30 works [5,6,12,15,28,29,30,31,32,33,34,35,36,37,38,39,40,41,42,43,44,45,46,47,48,49,50,51,52,53] focusing on fibrinogen modified either in vivo or in vitro (see Figure 2 and Table S1). The positions of PTMs are reported according to the mature chains, i.e., sequences without a signal peptide. There are two possible PTMs on 42 AAs, and three possible PTMs on 6 AAs. The Aα chain is most prone to PTMs (137 modified AAs at 109 sites), followed by the Bβ chain (105 AAs at 87 sites), and lastly, by the γ chain (65 AAs at 57 sites). Two phosphorylations and sulfations are reported in γ’, the minor form of the γ chain. No PTM is reported in the αE variant of the Aα chain, which may be explained by a low abundance of this form (~2%), thus the eventual PTMs are below the detection limit of mass spectrometry.

Positions of ten types of PTMs were reported: citrullination (reported in 7 works), followed by oxidation (7), phosphorylation (5), glycation (3), acylation (2; together with glycation), nitration (2), sulfation (2), homocysteinylation (2), hydroxylation (1) and glutamine cyclization (1). Oxidation is the most abundant PTM, affecting 113 AAs (44 in the Aα chain, 40 in the Bβ chain, and 29 in the γ chain). Citrullination is reported for 65 AAs (36/22/7), 46 AAs are glycated (22/17/7), 27 AAs are homocysteinylated (11/6/10), 21 AAs are nitrated (4/11/6), 17 AAs are acetylated (8/7/2), and 3 AAs are sulfated (0/1/2). Phosphorylation (12 AAs; 10/0/2) is recognized only in the Aα chain and in the C-terminal extension of the γ’ chain. The cyclization of BβQ1, the only cyclization known in fibrinogen, is the first PTM discovered in fibrinogen and the first AA cyclization reposted in a protein [49].

The position and type of PTMs, with the exception of the very first reports, were detected by mass spectrometry. Other works usually report the influence of an examined reactant or disease on fibrinogen properties. As there are many PTMs in the sample, it cannot be determined which of them cause alterations in fibrinogen behavior, and to what extent. For detailed information about the reported PTMs, see Table S1. Fibrinogen is co-translationally glycosylated at positions BβN364, γN52, and AαN667 [25,54], which are not included in our survey of PTMs.

Of all these PTMs, we chose AαR(Cit)104, γP(Ox)70, γK(Ox)75, γP(Ox)76, γP(Ox)76PGA, γN(Ox)77, and γM(Ox)78 to be characterized by MD simulations. These PTMs were chosen because they are at or close to the γ-coil, i.e., a region that was recently proposed [27] as able to interact with adjacent fibrinogen chains, changing their secondary structure. We aimed to gain further information about this phenomenon.

### 2.2. The Impact of PTMs on Secondary Structure

**Figure 3:**
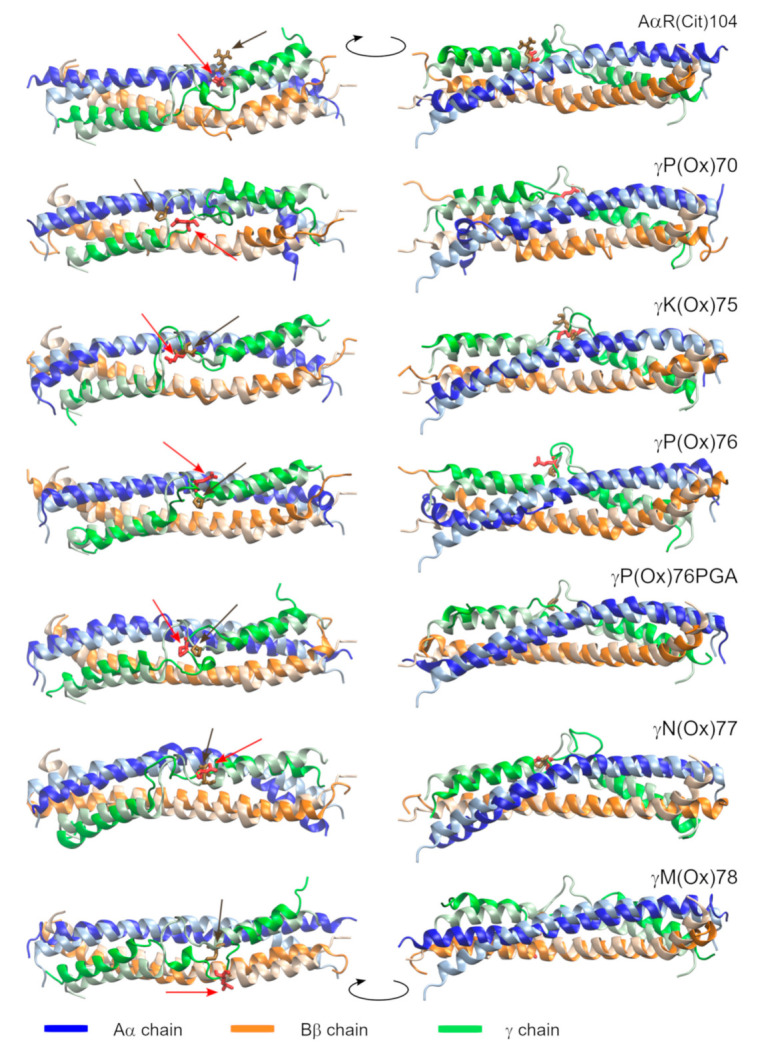
The geometry of the modified proteins (bright colors) fitted on the pristine fibrinogen (pale colors). The last frames of the MD simulation are shown. Modified AAs are shown in red and highlighted by red arrows. Their pristine counterparts are shown in brown and highlighted by brown arrows. N-termini are at the left part of the figure. The two images of each structure are rotated by 180° according to the axis perpendicular to the coiled-coil domain.

PTMs affect secondary structure in their vicinity in various ways, as demonstrated by the last frames of the MD simulations (Figure 3 and Figure S1). They can either fold the γ-coil (γY68 to γM78) or disturb the α-helices of Aα and Bβ chains by introducing π- or 3_10_-helices, coils or turns into their structure. The Kabsch–Sander classification [55], as implemented in DSSP [56], is used to characterize secondary structure development over time (Figure S2). The points of the secondary structure changes reported below are read out from these schemes. The individual systems are discussed below in detail.

Pristine fibrinogen preserves the structure of the Aα and γ chains (α-helix, resp. α-helix interrupted by a coil region). The AAs BβN135 to BβV139 switch between α- and π-helices from 47 to 111 ns (π-helix in 68.0% of analyzed frames; see Figure S2) and again from 140 to 207 ns (π-helix in 77.4% of frames). Another π-helix, containing the AAs BβV138 to BβY142, is formed between 210 and 240 ns (π-helix in 76.3% of frames). This simulation reveals that an interchange between α- and π-helices is a natural behavior of the coiled-coil domain of fibrinogen in the proximity of the γ-coil.

AαR(Cit)104 disrupts the α-helical fold of the AAs BβK133 to BβN140. Unfolding starts with the conversion of an α-helix to a 3_10_-helix at 4 ns. A bend followed by a turn is the dominant feature between 32 and 115 ns. Later, a 3_10_-helix becomes a dominant secondary structural element in this region (in 75.6% of the analyzed frames). Transitional π-helices were formed in the N-terminal direction of this interruption. The most prominent of them appears at 202 ns and contains the AAs BβW125 to BβQ129 (π-helix in 81.8% of frames). A turn occasionally switching to a 3_10_-helix (7.9%), followed by a bend (γE72 to γP76), is formed within the γ-coil after 102 ns.

γK(Ox)75 affects secondary structures in its vicinity in two ways. Firstly, it stabilizes the γ-coil by introducing a turn (71.6%) or bend (27.5%) made up of the AAs γ72 and γ73 at 60 ns. Secondly, a π-helix made up of the AAs BβK130 to BβN135 (88.7%) appears at 21 ns in the Bβ chain.

γP(Ox)76PGA results in the formation of a π-helix of AAs AαL94 to AαF98 (87.0%) at 82.5 ns. There are certain short-lived disturbances of the α-helix at different positions in the N-terminal direction of the π-helix. After 35 ns, the γ-coil is stabilized by a turn (87.8%), surrounded by bends that occasionally (7.6%) switch to a 3_10_-helix.

γP(Ox)76 is associated with the formation of a turn (74.0%) made up of the AAs AαA101 to AαN103, which appears at 177 ns. The γ-coil is stabilized by a turn (γD71 to γS73) after 106 ns.

γN(Ox)77 is manifested by the formation of a π-helix, made up of the AAs AαL94 to AαF98 (81.4%), at 25 ns.

γM(Ox)78 leads to the folding of the γ-coil (AAs γD71 to γS74) after 45 ns, when a 3_10_-helix appears. The 3_10_-helix turns into an α-helix at 175 ns. This process is associated with disturbances in the γ chain, as the AAs γQ65 to γT67 and γI79 to γT83 abandon their α-helical structure in favor of a turn at 234 ns resp. 240 ns.

The interpretation of the MD simulation describing γP(Ox)70 is not as straightforward as is the interpretation of the other simulations. This system exhibits a considerable number of artifacts resulting from the truncation of the coiled-coil domain of fibrinogen. The absence of such artifacts in other systems can on the other hand mean that the impact of γP(Ox)70 on fibrinogen is great enough to let these artifacts show. Thus, the impact of γP(Ox)70 on fibrinogen is considerably greater than the effect of the other PTMs described in this report. Analysis of the development of secondary structures in time shows a coil region in the Bβ chain (BβE141 and BβY142) since the beginning of the simulation. This disturbance of the α-helical structure extends by an N-terminal π-helix (BβE136 to BβN140) at 75.5 ns. Another π-helix, formed by the AAs BβK122 to BβQ126, appears at 12 ns. The AAs BβY119 to BβL121 abandon their α-helical structure at 16 ns, switching to either a turn (61.8%), 3_10_-helix (29.2%), coil (6.8%), or α-helix (6.1%). The Aα chain is disturbed after the first frames of the simulations. The AAs AαR104 to AαV111 switch between 3_10_- and π-helices and a turn. A coil containing AαV111 and AαS112 appears at 114 ns, and results in a 90°-bend in the Aα chain (Figure 3 and Figure S1). This would not have occurred had the system been linked by the disulfide bridges.

Figure S2 shows that the γ-coil is prone to secondary structure changes. For this reason, we performed further analyses of this region. The average number of AAs adopting a given secondary structure (Table 1) are computed over the last 50 ns of trajectories, when most of the systems are equilibrated. The length of γ-coil varies from 9 (γP(Ox)76) to 19 (γM(Ox)78) AAs, with a mode value of 11 AAs.

The most abundant (6.0 to 8.8 AAa) secondary structure element of the γ-coil is, predictably, a coil; i.e., AAs do not adopt any secondary structure. Other AAs adopt either a turn or a bend. Both secondary structures are stabilized by a single hydrogen bond, and they differ in the angle adopted by the protein backbone. If the angle is greater than 70°, the structure is classified as a bend, otherwise, it is a turn [55]. γM(Ox)78 is the only system forming an α-helix in the γ-coil. The 3_10_-helices and β-bridges sporadically appear in the γ-coil.

Changes of the structure at the termini of the chains will not be discussed here, because they originate from the artificial interactions at the ends of the chains and would not have occurred in full-length systems.

### 2.3. The Impact of PTMs on the Geometry of Protein

**Figure 4:**
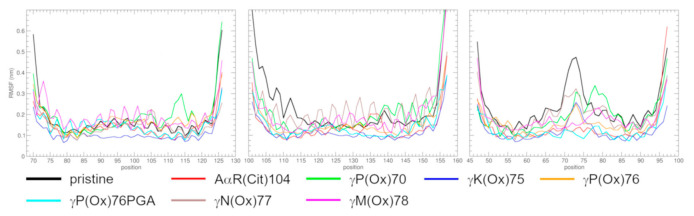
RMSF over the last 50 ns of MD simulations.

The root mean square deviation (RMSD; Figure S3), root mean square fluctuation (RMSF; Figure 4), and radius of gyration (RG; Figure S4) of Cα carbons are computed to see the subtle differences in fibrinogen geometry. The averages shown in Table 2 are computed over the last 50 ns of simulations. The structures before geometry optimization are used as a reference for RMSD and RG calculations, to be able to compare the extent of geometry changes in the systems. RMSF uses the average position of a particle as a reference. Let us remember that RMSD characterizes the time-dependence of deviation of protein geometry with respect to the initial structure. RMSF refers to the extent of particle fluctuation from its average position over time (here, 50 ns). RG is linked with the compactness of protein.

The RMSD of γP(Ox)70 and γM(Ox)78 does not oscillate around an equilibrium value (Figure S3). This means that their geometry did not yet reach the (local) minimum, and their development is ongoing. The equilibration of the RMSD of AαR(Cit)104 and γN(Ox)77 is questionable. This information needs to be considered when interpreting the results. All systems, on the other hand, reached an energy equilibrium (Figure S4).

The overall RMSD of PTM-containing systems reached a higher value than that of pristine fibrinogen. This tells us that the modified systems are more remote from the initial geometry than pristine fibrinogen. Systems describing modified proline reach the highest values of RMSD, and γM(Ox)78 and γK(Ox)75 the lowest values, almost on the level of pristine fibrinogen.

The RG is computed to see if changes in RMSD are caused by variations in secondary structure, or if they point to changes in protein dimensions. The RG is, in all systems, smaller than in the crystal structure, which points to a decrease in protein dimensions. A decrease within 10% of the value obtained from crystal structure excludes the collapse of the protein, and is explained by the bending of the coiled-coil region, as was previously observed [19,27].

The RMSF (over the last 50 ns of simulations) is computed to quantify the movement of individual AAs, here represented by their Cα carbons. Pristine fibrinogen is used as an example for a description of the curves. Biases from this behavior are highlighted for the modified systems.

The RMSF of pristine fibrinogen (Figure 4) shows a saw-like shape typical in α-helices. The π-helix observed in the Bβ chain induces a peak around BβV139 into the curve. The dominant feature of the γ chain is a peak in the middle of the curve (AAs γY68 to γP76), which represents the flexible γ-coil. A high RMSF at the ends of the chains represents the unfolded termini.

The Aα chain preserves its α-helical structure over the whole course of the studied fragment except for γP(Ox)70, which exhibits a peak around AαD114. This peak corresponds to the above-mentioned coil and is an artifact. A smaller peak is centered around AαS117 in γP(Ox)76, which results from the bend. Note also the relatively low RMSF of γK(Ox)75, and high amplitude in γM(Ox)78. The high amplitude means that there is a tension in the system which could be relaxed by some conformation changes that would occur had the simulation been longer.

The RMSF of the Bβ chain resembles that of the Aα chain. It depicts the α-helical structure of the chain with local disturbances in curves representing AαR(Cit)104 and γP(Ox)70. These slightly higher regions represent a 3_10_-helix resp. turn. Another interesting feature in this graph is the high amplitude in the γK(Ox)75 system, which usually precedes structural changes in the system.

The most prominent feature in the RMSF of the γ chain is a peak centered around γS73. This peak is lower in modified molecules than in pristine fibrinogen, which means that their γ-coil is less flexible. The peak is absent in AαR(Cit)104 and γP(Ox)76PGA. The center of the curve is low for AαM(Ox)78, and its maximum is located at γI79, the N-terminal AA of the C-terminal extension of the γ-coil. γP(Ox)70 also exhibits the irregular shape of the central peak, with a maximum at γM78 and a depression at γK75.

## 3. Discussion

MD simulations describing the impact of AαR(Cit)104, γP(Ox)70, γK(Ox)75, γP(Ox)76, γP(Ox)76PGA, γN(Ox)77 and γM(Ox)78 on fibrinogen structure are reported, and their behavior is compared with MD simulations of pristine fibrinogen. For the possible limitations of this approach, see [27]. When interpreting the results, one must bear in mind that the geometry of systems γP(Ox)70 and γM(Ox)78 did not reach equilibrium, and that the equilibrium reached by systems AαR(Cit)104 and γN(Ox)77 is doubtful. This means that the development of these systems is ongoing, and we do not see the resultant state after the introduction of PTMs.

Systems containing PTMs can be considered as special cases of pristine fibrinogen, where PTM may induce certain behavior that would, in pristine fibrinogen, occur with a lower probability or at longer timescales than in PTM-containing systems. Events occurring in several systems may be the general characteristics of the protein and not the general consequences of PTMs.

One such feature is switching between α- and π-helices. It appears in all systems at similar positions (Bβ130–Bβ142 resp. Aα94–Aα98) that are close to plasmin cleavage sites. For π-helices that facilitate the binding of enzymes to proteins [57], PTMs may influence their position and frequency of appearance. For most of the examined PTMs that are in the γ-coil, we hypothesize that the γ-coil affects the formation of π-helices, probably by its interactions with other chains. Simulations predict that γK(Ox)75, γP(Ox)76PGA, and γN(Ox)77 stabilize the newly-formed π-helices, and that γM(Ox)78 restrains its formation.

Apart from π-helices, certain segments of α-helices switch into a 3_10_-helix (AαR(Cit)104), turn (γP(Ox)76), and coil (γP(Ox)70). Excluding the coil that results from truncation of the coiled-coil domain (see Results), we consider the 3_10_-helix and turn a consequence of PTM.

Another feature that is common for all PTM-containing systems reported in this study is a decrease in fluctuations of the γ-coil in comparison with the pristine fibrinogen. This is explained by the formation of stable secondary structure elements within the γ-coil. The presence of even a single hydrogen bond that defines a bend and turn is effective in decreasing γ-coil flexibility. This finding is confirmed by an absence of stable secondary structures in pristine fibrinogen, γN(Ox)77, γP(Ox)70, γK(Ox)75, and γP(Ox)76, i.e., systems showing a higher RMSF of the γ-coil. It is questionable whether this behavior can be considered a general characteristic of modified fibrinogen, since other simulations [27] showed that PTMs can increase the level of fluctuation of the γ-coil. To be able to compare RMSFs reported in two studies, we computed an additional RMSF for the γ chain from 75 to 100 ns (Figure S6), as computed in the previous study. The graph shows that the decrease in the RMSF is considerably smaller for γM(Ox)78 than for RMSFs computed between 200 to 250 ns. This is interpreted as showing that the secondary structure within the γ-coil needs some time to establish, and once it is established, it decreases the fluctuation of the γ-coil.

Various PTMs alter fibrinogen behavior to a different extent and manner, as was reported from earlier experiments. We performed MD simulations to shed light on these changes at an atomistic level. Below, we discuss the effects of examined PTMs in the context of experimentally observed changes in fibrinogen behavior. Note that the samples contain other PTMs than those which participated in the reported alterations. The presented simulations do not distinguish between fibrinogen (which contains fibrinopeptides and does not polymerize) and fibrin (which does not contain fibrinopeptides and will polymerize), as the region where the two forms vary is absent from our simulations. We believe that the results are valid for both fibrinogen and fibrin.

Citrullination of fibrinogen by enzyme peptidyl arginine deaminase 2, but not by peptidyl arginine deaminase 4, inhibits fibrin polymerization and decreases the rate of protofibril formation, the thickness of fibrin fibers, and overall hemostatic potential [58]. Changes in the characteristics associated with the rate of polymerization are explained by the citrullination of the arginines AαR16 and BβR14, which block fibrinopeptide cleavage by thrombin [16]. AαR(Cit)104 may participate in decreasing fibrin fiber thickness. The nature of this effect remains unknown, as the formation of fibrin fiber is not yet understood. It should be mentioned that the position and properties of this PTM were determined in different studies [15,58]. This does not necessarily mean that the PTM detected in one out of four rheumatoid arthritis patients is not present in samples citrullinated in vitro. It should be considered that there are other citrullinations in the characterized samples that can participate in the alteration of fibrinogen properties. Apart from Sharma et al. [15], this PTM was reported by van Beers et al. [35], and by a Japanese group who lately stated that they misinterpreted the data, and that the signal detected by mass spectrometry belongs to an adjacent deamidation [32,33].

The oxidation of γP70 to glutamic semialdehyde, among other PTMs, was reported in fibrinogen that was oxidized in vitro by O_3_ [29]. No effect of oxidation on fibrinogen properties is described in the cited paper, but it can be found in the paper by Rosenfeld et al. [13]. It is questionable whether γP(Ox)70 is present in the samples characterized by the latter work, as it is not reported in a paper by Yurina et al. [30] which extends the work of Bychkova et al. [29]. Both reports originate from the same group and use the same setup for their experiment. This may also mean that γP(Ox)70 is a rare PTM. The ozone-induced oxidation of fibrinogen is reported to speed up fibrinogen cross-linking by factor XIIIa, and to decrease the number of α-helices in fibrinogen. The γP(Ox)70 is highly unlikely to influence fibrin cross-linking, because the coiled-coil domain does not cross-link. The MD simulation of this PTM revealed changes in fibrinogen’s secondary structure, including the unfolding of α-helices in the coiled-coil region.

The oxidation of fibrinogen by hypochlorite (ClO^−^) induced, inter alia, oxidation of γK(Ox)75, γP(Ox)76, and γN(Ox)77 [12]. This study reports that oxidation of fibrinogen by 50 μmol HOCl/mg fibrinogen (i.e., the concentration used for PTMs detection) does not cause fibrinogen fragmentation, but it does inhibit fibrinogen polymerization and decreases its turbidity. The latter is explained by the formation of a fibrin clot with smaller pores and thinner fibers than that formed of pristine fibrin [26]. Regarding their position in the coiled-coil domain, these PTMs should not affect fibrin polymerization, although they could influence fibrin-clot properties by altering fibrinogen structure and behavior. Of these three PTMs, the impact of γP(Ox)76 on fibrinogen behavior seems to be more significant than the effect of γK(Ox)75 and γN(Ox)77, because it results in the formation of a turn in the Aα chain. The other two PTMs induce π-helices in the coiled-coil domain. As switching between π- and α-helices is observed in pristine fibrinogen, we do not expect that it could noticeably influence the architecture of a fibrin clot. We hypothesize that the newly formed π-helices are more stable than those of the pristine fibrinogen, which could influence the rate of fibrinolysis by plasmin.

γP(Ox)76PGA is reported to result from fibrinogen oxidization by ClO^−^ or malondialdehyde [27,59]. Fibrin modified by both reagents forms thicker fibers than pristine fibrin. While malondialdehyde-treated fibrinogen forms denser clots, clots formed from fibrinogen oxidized by ClO^−^ are sparser and contain knobs. The study reports many other PTMs than γP(Ox)76PGA. The MD simulation shows that γP(Ox)76PGA has little effect on fibrinogen; namely, it induces a π-helix in the Aα chain and it stabilizes the γ-coil. We do not expect that any of these alterations affect the architecture of fibrin clots.

The difference in the behavior of γP(Ox)76 and γP(Ox)76PGA is likely caused by the nature of the introduced residues. While pyroglutamic acid is cyclic, like proline, glutamic semialdehyde is linear.

The oxidation of γM78 to methionine sulfoxide is reported in fibrinogen oxidation by ClO^−^ [5,12,28], O_3_ [29,30], and H_2_O_2_ [31]. Other PTMs are mentioned in all reports. Other works dealing with fibrinogen oxidation by ClO^−^ report decreased rates of polymerization and turbidity. Weigandt et al. [5] further report thinner fibrin fibers, decreased clot permeability, decreased clot lysis, and increased clot density. An increased concentration of the oxidizing agent increases levels of γM78 oxidation. The O_3_ oxidation of fibrinogen decreases the content of α-helices in protein [13]. The oxidation by H_2_O_2_ decreases fibrin cross-linking and the stiffness of the clot [31]. The most significant manifestation of γM(Ox)78 at an atomistic level is a formation of an α-helix within the γ-coil. The γ-coil consequently extends in both directions. There are no stable π-helices in Aα and Bβ chains, which may point to the association of a secondary structure in the γ-coil with disturbances in the α-helical structure of the other chains. Methionine sulfoxide exists in two diastereomers, with sulfur as a chiral center [60]. For simplicity, we considered only the S isomer, and expect that its R counterpart will behave in the same manner.

## 4. Materials and Methods

The PTMs of fibrin and fibrinogen were searched in the literature using the scholar.google and PubMed [20] web interfaces. Only works reporting a new position or type of PTM were considered. PTMs in the γ-coil or in adjacent regions of the Aα and Bβ chains were chosen for MD simulation. The positions of PTMs are referred for mature chains of fibrinogen (i.e., sequences do not contain signal peptides).

The initial geometry of the central part of the coiled-coil domain of fibrinogen was obtained by adjusting the crystal structure 3GHG [18] to contain AAs 70 to 126 of the A chain, 101 to 157 of the B chain, and 47 to 97 of the C chain. PTMs were introduced into structures by the Vienna-PTM 2.0 web server that changes the geometry of the newly-induced AA and subsequently performs steepest descent geometry optimization [61]. For pristine fibrinogen, γP(Ox)76, and γP(Ox)76PGA, our previously reported simulations [27] were extended from 100 to 250 ns.

MD simulations were performed using the Gromacs package [62], with a Gromos 54a7 force field [63,64]. Production simulations with the timestep of 2 fs were performed for 250 ns at 310 K and 1 bar. For a detailed description of the simulation protocol, see Text S1 in the Supplementary Materials. Analyses were performed by standard Gromacs tools, using the DSSP utility for secondary structure determination. Graphs were prepared with xmgrace, and protein structures were visualized by VMD [65]. RMSF and the averages of RMSD and RG were computed over the last 50 ns of the simulation. Secondary structure analyses were computed from a trajectory containing every 50th frame of the simulation.

## 5. Conclusions

The study describes the impact of seven PTMs on the behavior of the fibrinogen coiled-coil domain at the atomistic level. The examined PTMs were chosen from a list of 307 PTMs reported in the literature.

We showed that certain PTMs can destabilize the α-helical structure of the coiled-coil domain of fibrinogen by their conversion to a coil, turn or 3_10_-helix, and that folding of the γ-coil into α-helix decreases the content of π-helices in Aα and Bβ chains. The structure of Aα and Bβ chains is not strictly α-helical, for its segments switch into short π-helices (usually of 5 AAs, i.e., one π-helical turn). The position of these π-helices may vary within a single system. Such behavior is observed even in pristine fibrinogen. No π-helices were observed in the examined part of the γ chain.

As π-helices facilitate the binding of enzymes, and the reported π-helices are located near the plasmin cleavage site, we hypothesize that they may facilitate fibrin(ogen)–plasmin interactions. Furthermore, we found that the properties of these π-helical segments are influenced by the behavior of the γ-coil. This information is not only interesting from the viewpoint of fundamental research, for it proposes that problems with fibrinolysis may be treated by alteration of the behavior of the γ-coil, for instance by a drug. Alternatively, the application of a ROS or other PTM-inducing reactive metabolites into the system might be used to adjust the fibrinogen behavior they promote when this would be desirable from a therapeutic point of view.

## Figures and Tables

**Table 1:** Range of γ-coil and secondary structure elements within the γ-coil over the last 50 ns of the simulation.

	Range	Coil	Bend	Turn	α-Helix	3_10_-Helix	β-Bridge
pristine	γY68–γM78	7.4 ± 1.1	2.7 ± 1.7	0.9 ± 1.0			
AαR(Ox)104	γY68–γM78	6.0 ± 3.0	2.4 ± 0.8	2.4 ± 1.0		0.3 ± 0.9	
γP(Ox)70	γY68–γN77	7.1 ± 1.2	2.0 ± 1.1	0.1 ± 1.0			0.1 ± 0.4
γK(Ox)75	γY68–γM78	8.8 ± 0.6	0.9 ± 1.2	1.3 ± 1.0			
γP(Ox)76	γN69–γN77	6.0 ± 0.2	0.1 ± 0.5	2.9 ± 0.7		0.1 ± 0.4	
γP(Ox)76PGA	γT67–γA81	8.1 ± 0.9	4.0 ± 1.0	2.8 ± 0.8		0.1 ± 0.6	
γN(Ox)77	γY68–γI79	8.0 ± 0.7	1.2 ± 0.8	2.8 ± 0.7		0.1 ± 0.5	
γM(Ox)78	γQ65–γT83	6.2 ± 1.1	5.4 ± 1.0	0.3 ± 0.9	7.0 ± 2.2	0.1 ± 0.6	

**Table 2:** Averages of RMSD and RG over the last 50 ns of the simulations.

	RMSD [nm]	RG [nm]
pristine	0.413 ± 0.038	2.385 ± 0.028
AαR(Ox)104	0.499 ± 0.039	2.322 ± 0.018
γP(Ox)70	0.708 ± 0.054	2.253 ± 0.033
γK(Ox)75	0.449 ± 0.032	2.358 ± 0.016
γP(Ox)76	0.602 ± 0.054	2.327 ± 0.027
γP(Ox)76PGA	0.557 ± 0.031	2.377 ± 0.020
γN(Ox)77	0.494 ± 0.041	2.372 ± 0.021
γM(Ox)78	0.440 ± 0.046	2.452 ± 0.036
3GHG		2.460

## Data Availability

The data presented in this study are available in the article and Supplementary Materials.

## Supplementary Materials

The following are available online at https://www.mdpi.com/article/10.3390/metabo11050307/s1, Table S1: PTMs within fibrinogen, Figure S1: An alternative representation the protein geometries, Figure S2: Development of secondary structure in time, Figure S3: Development of RMSD in time, Figure S4: Development of RG in time, Figure S5: Development of energy in time, Figure S6: RMSF of the γ chain computed between 75 and 100 ns, Text S1: Detailed description of simulation settings.
