# Supplementary material for: Molecular Dynamic Simulations Suggest That Metabolite-Induced Post-Translational Modifications Alter the Behavior of the Fibrinogen Coiled-Coil Domain
Source: Metabolites. 2021 May 11;11(5):307. doi: 10.3390/metabo11050307 (PMC8150326; doi:10.3390/metabo11050307)
Supplement: Supplementary file 1 [file metabolites-11-00307-s001.zip › SI.pdf]

Figure S1: Protein geometry of the last frames of the MD simulation. Modified AAs are highlighted by arrows. AαR104 is shown in orange, γP70 in pink, γK75 in red, γP76 in green, γN77 in cyan, and γM78 in brown.

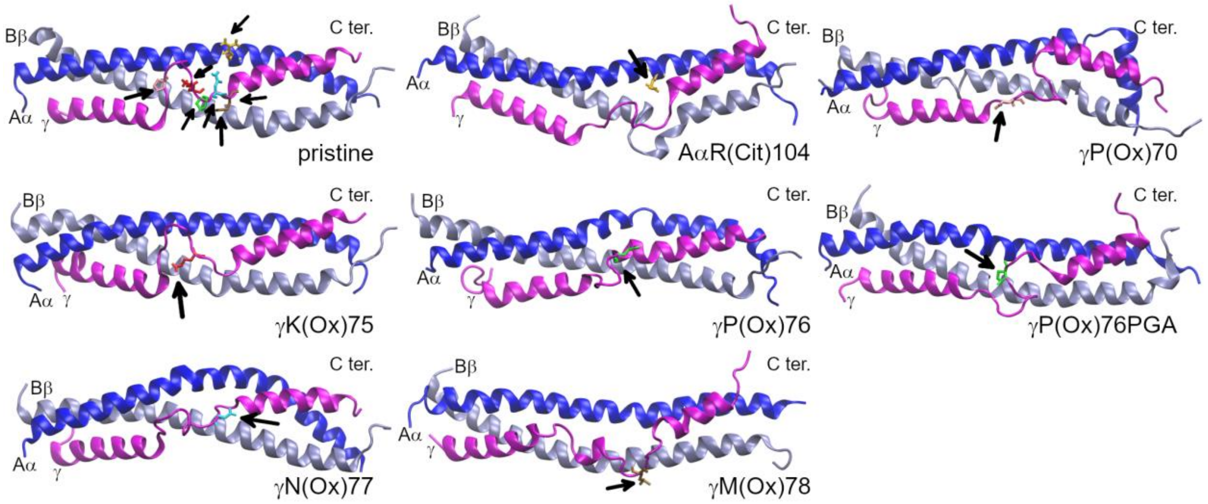

Figure S2 (next 8 pages): Development of secondary structure in time for all examined systems.

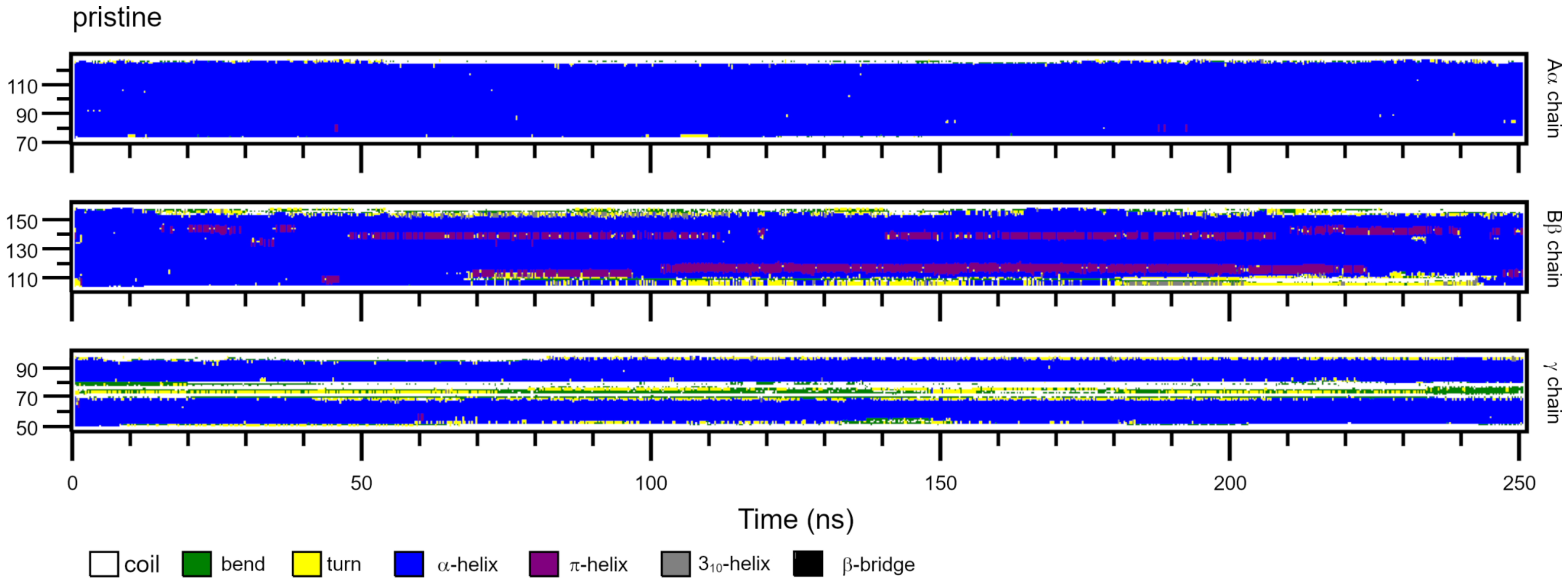

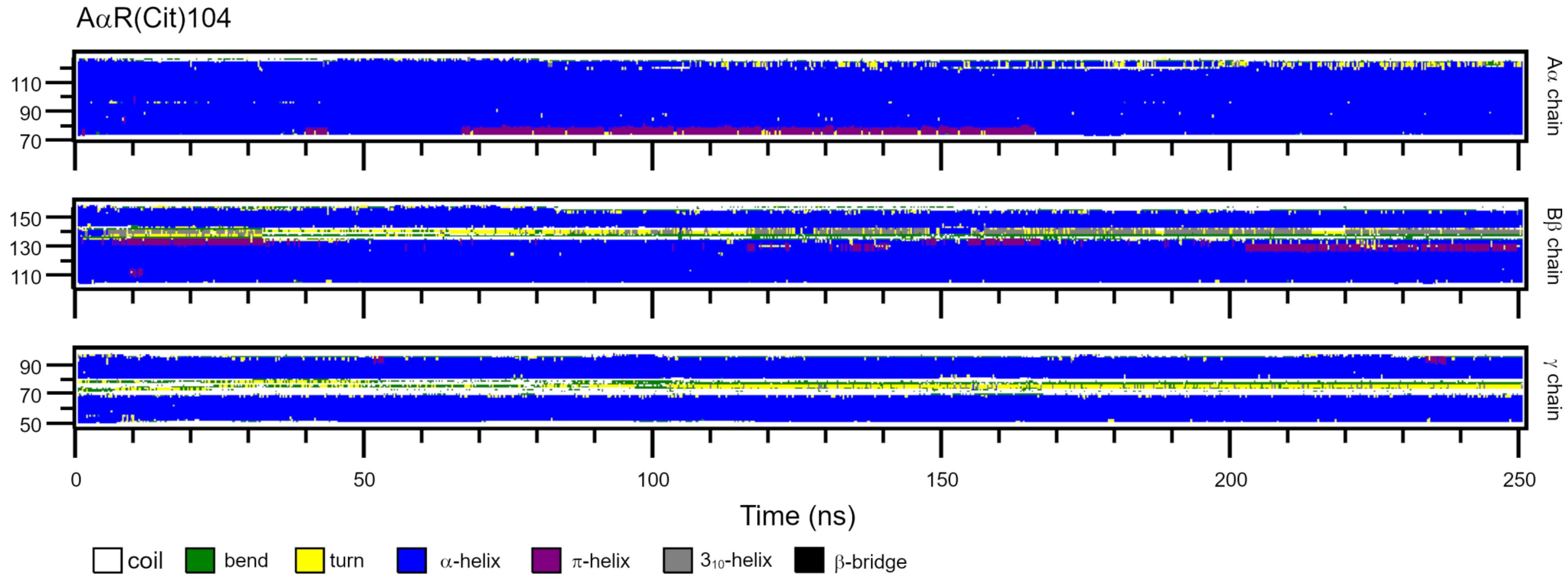
AαR(Cit)104
110
90
70
Aα chain
150
130
110
Bβ chain
90
70
50
γ chain
0
50
100
150
200
250
Time (ns)
coil
bend
turn
α-helix
π-helix
3₁₀-helix
β-bridge

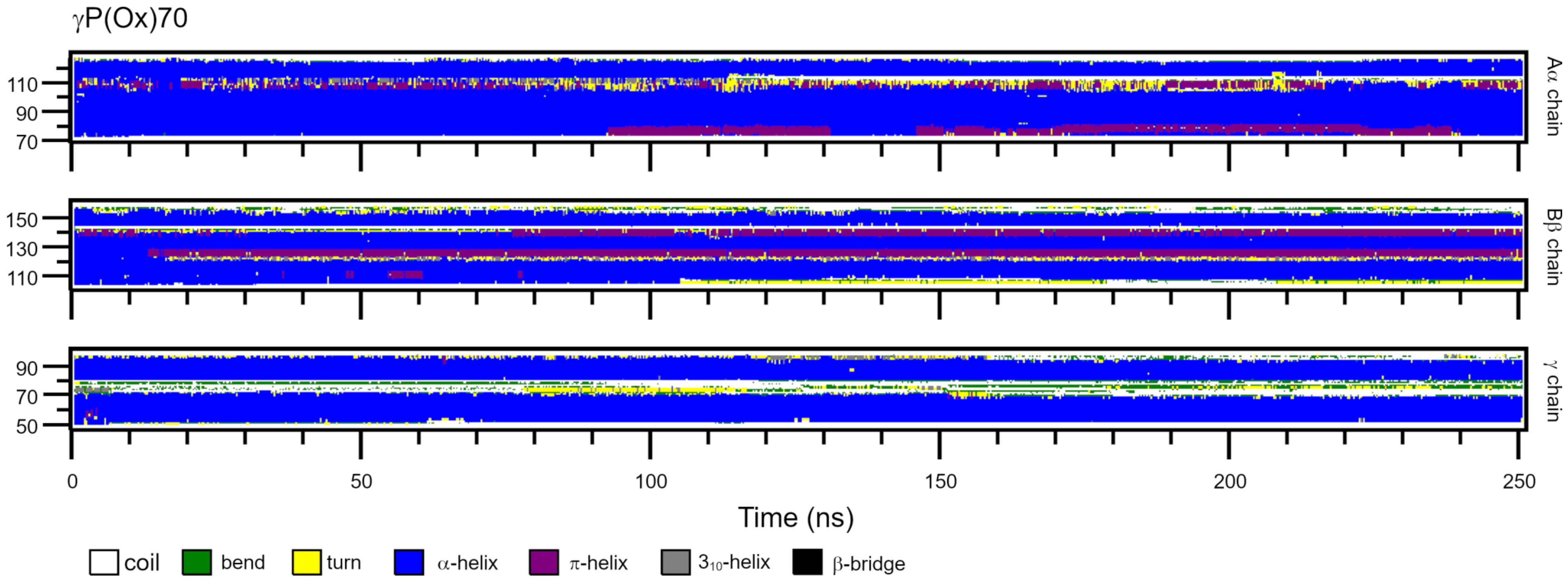
γP(Ox)70
Aα chain
Bβ chain
γ chain
110
90
70
150
130
110
90
70
50
0
50
100
150
200
250
Time (ns)
coil
bend
turn
α-helix
π-helix
3₁₀-helix
β-bridge

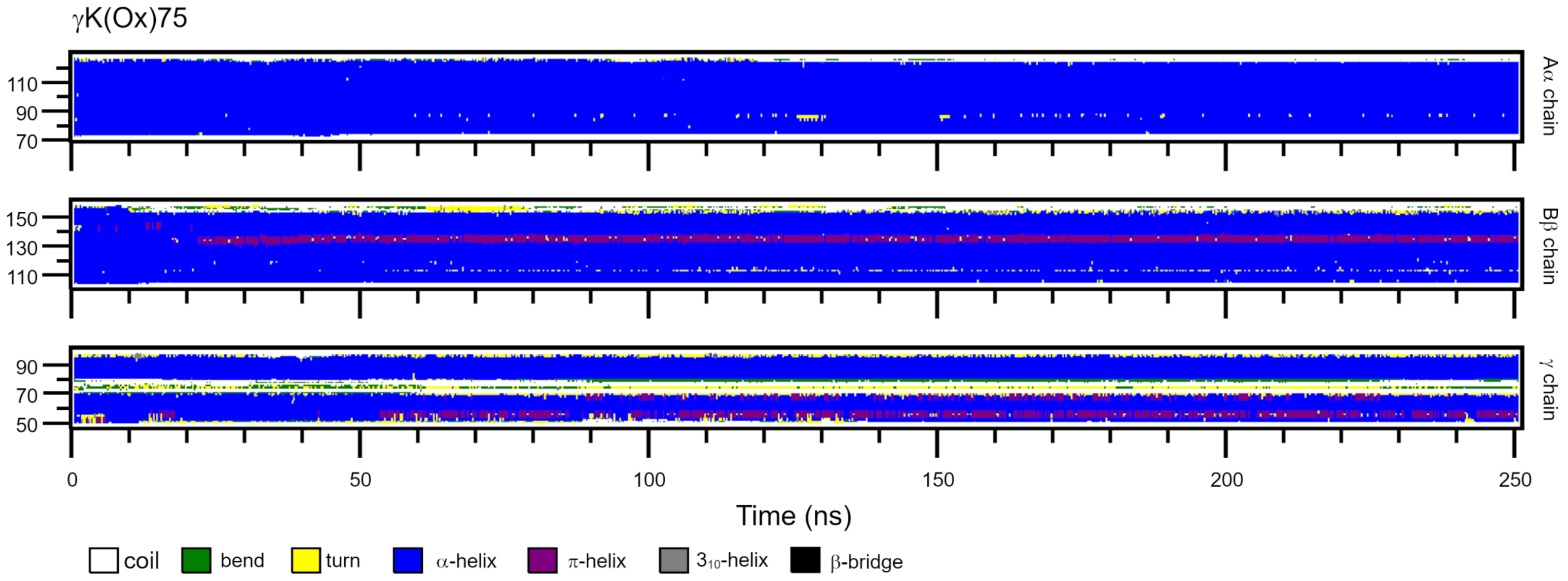

γK(Ox)75
Aα chain
Bβ chain
γ chain
110
90
70
150
130
110
90
70
50
0
50
100
150
200
250
Time (ns)
coil
bend
turn
α-helix
π-helix
3_{10}-helix
β-bridge

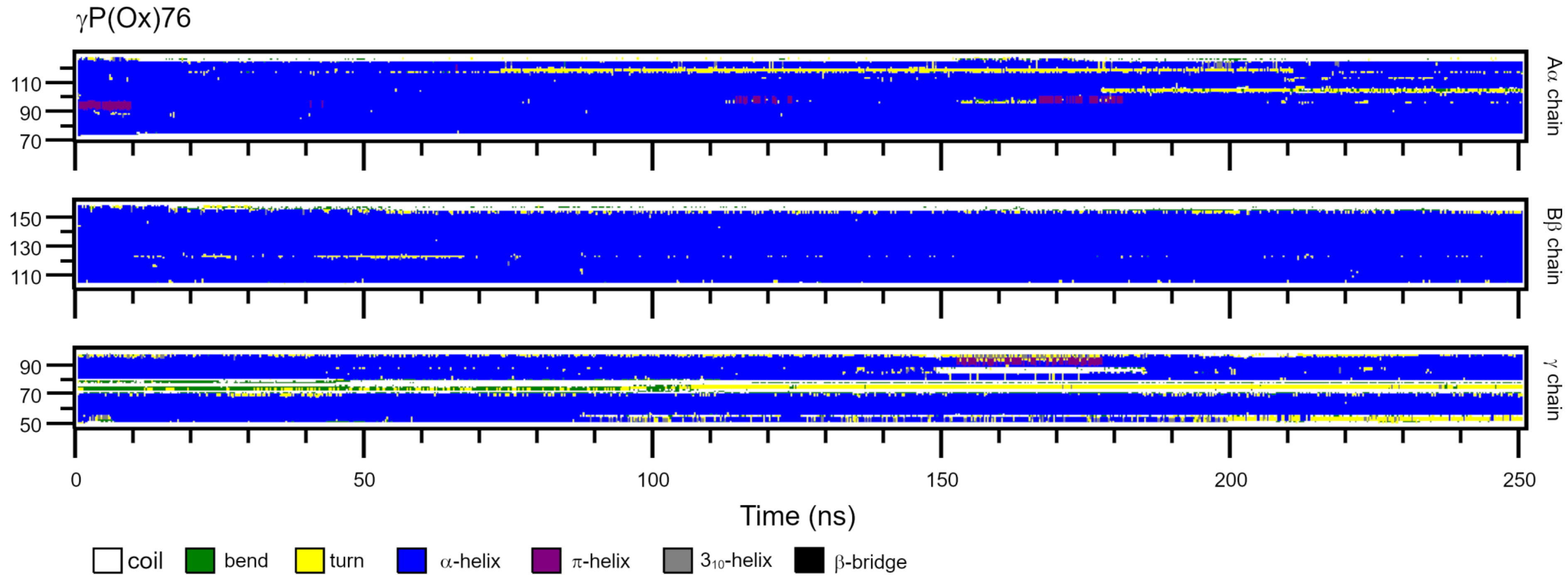
γP(Ox)76
Aα chain
Bβ chain
γ chain
70
90
110
110
130
150
50
70
90
0
50
100
150
200
250
Time (ns)
coil
bend
turn
α-helix
π-helix
3₁₀-helix
β-bridge

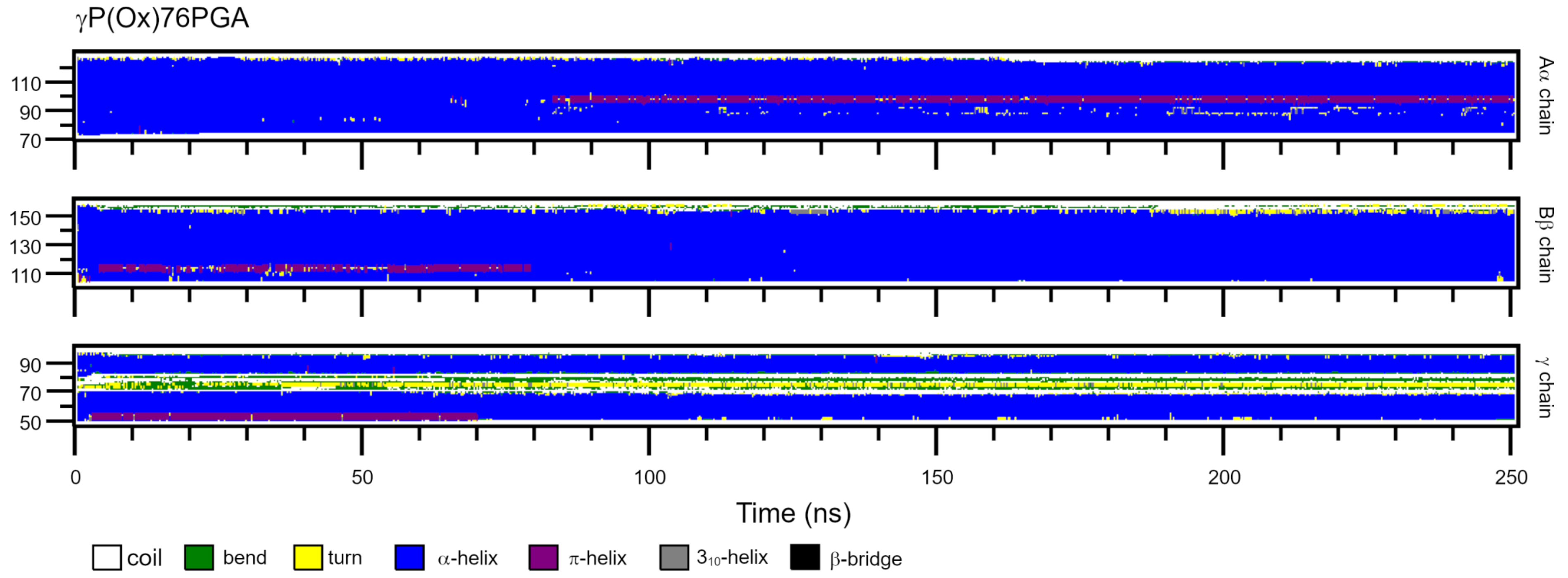
γP(Ox)76PGA
110
90
70
Aα chain
150
130
110
Bβ chain
90
70
50
γ chain
0
50
100
150
200
250
Time (ns)
coil
bend
turn
α-helix
π-helix
3₁₀-helix
β-bridge

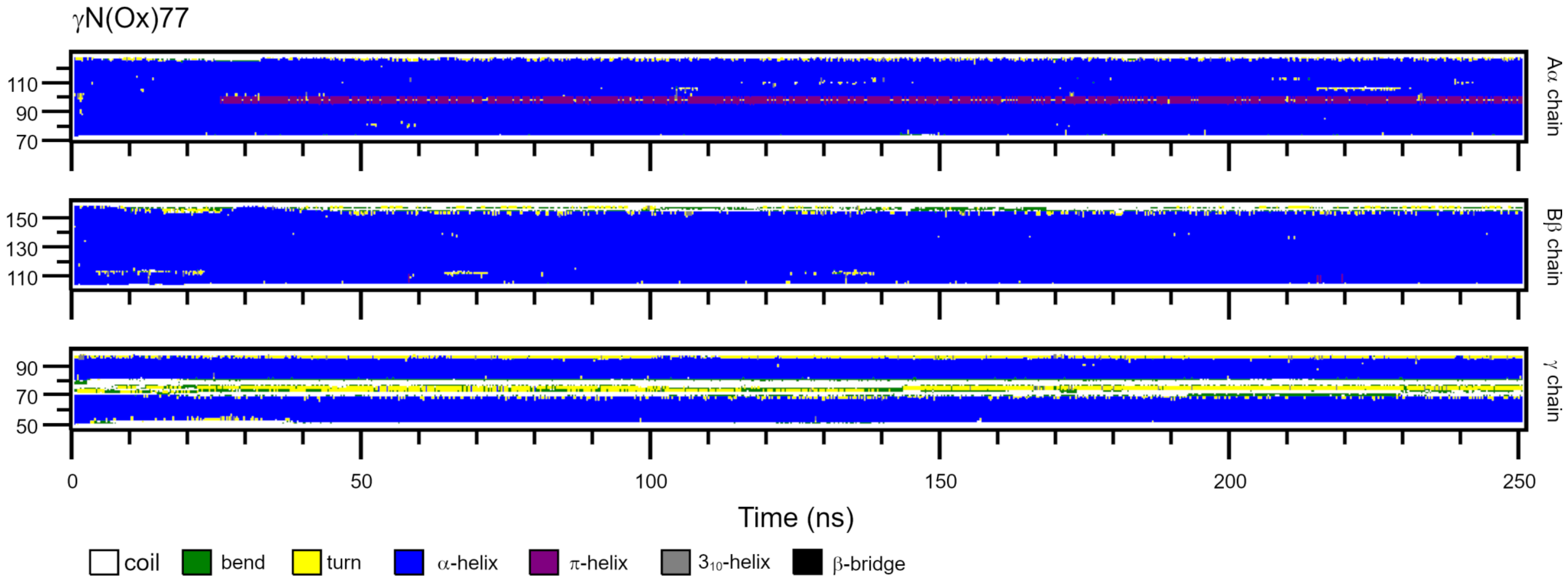

γN(Ox)77
110
90
70
Aα chain
150
130
110
Bβ chain
90
70
50
γ chain
0
50
100
150
200
250
Time (ns)
coil
bend
turn
α-helix
π-helix
3₁₀-helix
β-bridge

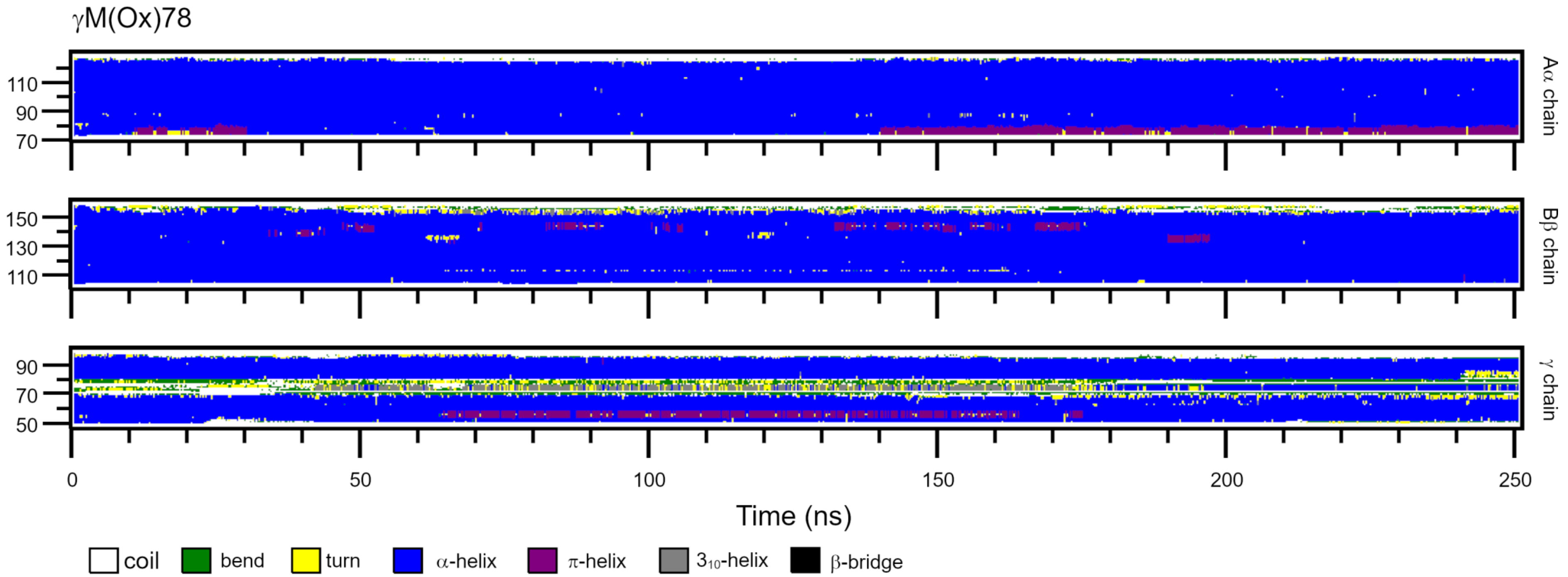

γM(Ox)78
110
90
70
Aα chain
150
130
110
Bβ chain
90
70
50
γ chain
0
50
100
150
200
250
Time (ns)
coil
bend
turn
α-helix
π-helix
3₁₀-helix
β-bridge

Figure S3: Development of RMSD in time for C_α carbons of examined systems.

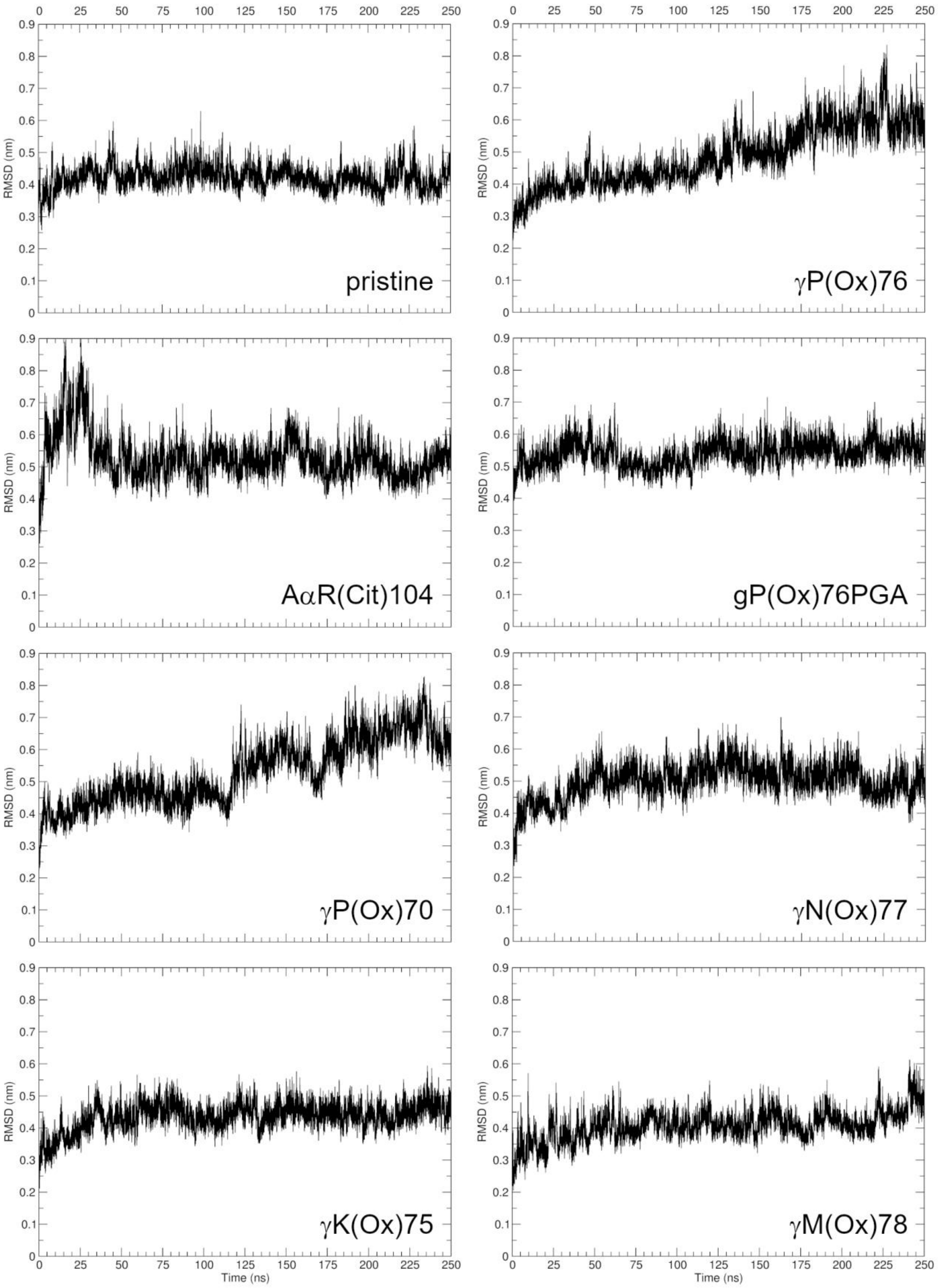

Figure S4: Development of RG in time for C_α carbons of examined systems.

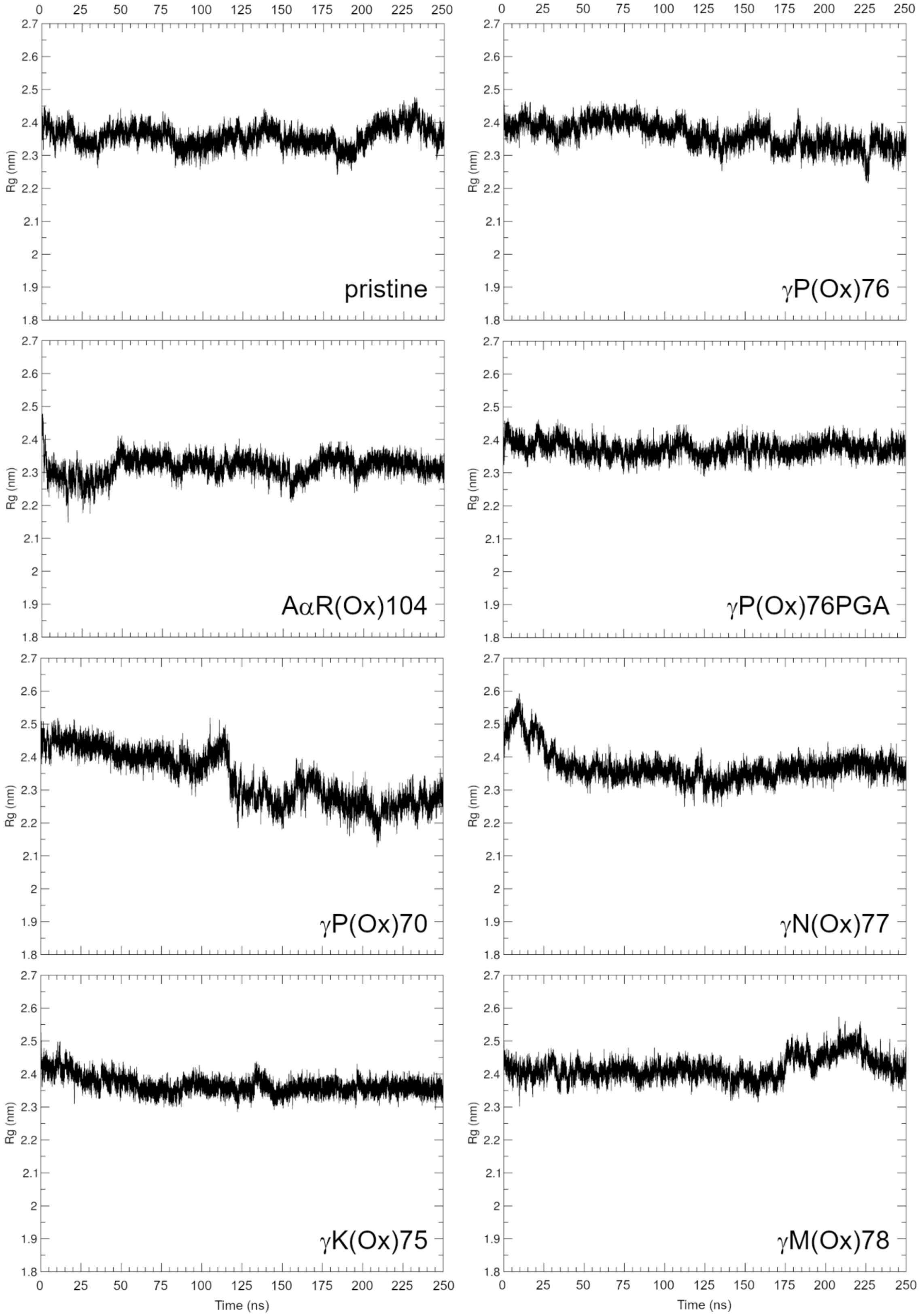

Figure S5: Development of the energy of the system in time. Potential energy is shown in black, kinetic energy in red and total energy in green. The tiny decrease of energies at 100 ns in the γP(Ox)76PGA origins in the way of extending this system from 100 to 250 ns.

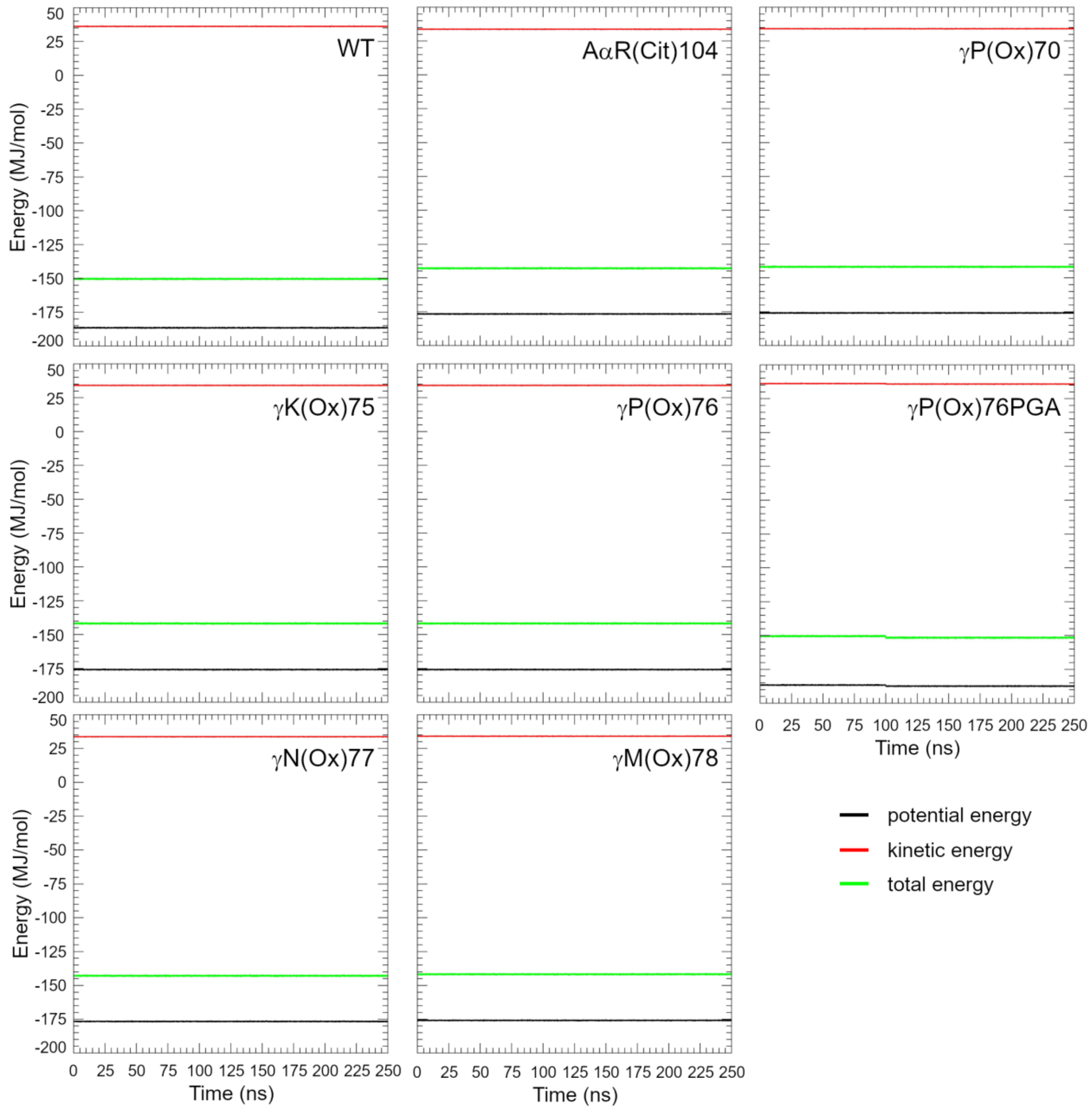

Figure S6: RMSF of the γ chain computed between 75 and 100 ns.

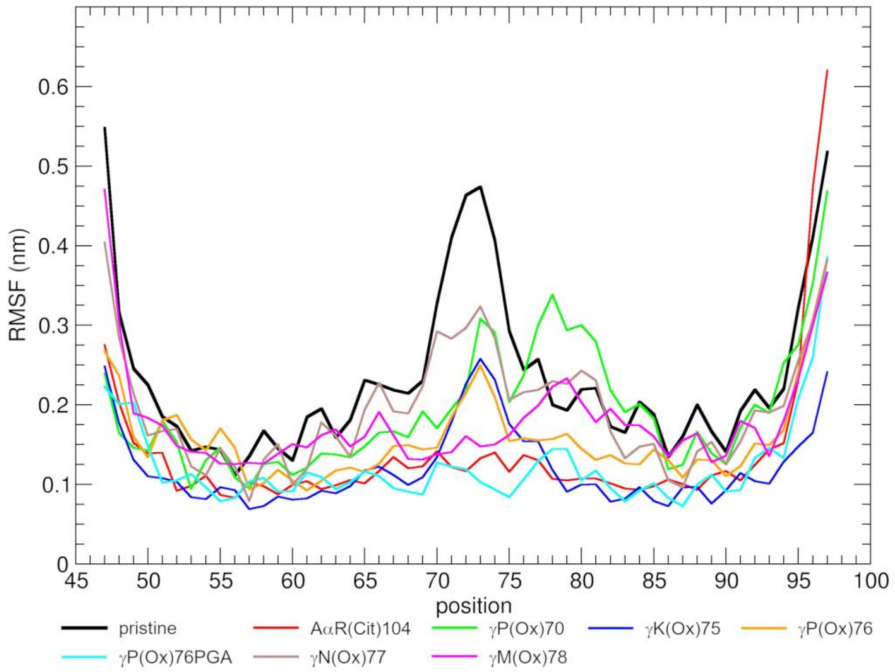

Text S1: Detail description of the simulation protocol

This study is performed on central part of fibrinogen coiled-coil domain that is represented by amino acids 70 to 126 of the A chain, 101 to 157 of the B chain and 47 to 97 of the C chain of crystal structure 3GHG [1]. Post-translational modifications were introduced into the structures by Vienna-PTM 2.0 web server [2]. Systems WT, γP(Ox)76 and γP(Ox)76PGA represent extension of our previously reported simulations [3] from 100 to 250 ns. Molecular dynamic (MD) simulations were performed by Gromacs package [4] with Gromos 54a7 force field [5,6]. Production simulations with the timestep of 2 fs were performed for 250 ns and frames were written every 250 ps. Protein was solvated by SPC water [7], if necessary counterions were added. Temperature was hold at 310 K by Bussi's velocity rescaling thermostat [8] that was independently connected to protein and water (with counterions) once a picosecond. Pressure was hold on 1 bar by Berendsen barostat [9] with compressibility of 4.5×10^{-5} bar^{-1}, that was connected to the system in isotropic manner once 5.1 ps. Long-range interaction was cut at 1.4 nm and Coulombic interactions were further treated by reaction field approximation (ε_r = 65) [10]. Neighbor list starts at 1.4 nm and was updated every 10 steps. Long range dispersion correction was applied for energy and pressure. All bonds were constrained by the 4-order LINCS algorithm [11]. Prior the production simulation, geometry of systems was optimized by 10 steepest descent optimization runs each of 10 steps that was followed by 50 ps MD simulation with the timestep of 1 fs with restrain of 1000 kJ/mol nm^2 applied on protein and another 50 ps MD simulation with the timestep of 1 fs without position restrains. All other settings were identical to the production simulation.

References:

[1] Kollman JM, Pandi L, Sawaya MR, Riley M, Doolittle RF. Crystal structure of human fibrinogen. Biochemistry 2009;48:3877-86.

[2] Margreitter C, Petrov D, Zagrovic B. Vienna-PTM web server: a toolkit for MD simulations of protein post-translational modifications. Nucleic Acids Res 2013;41:W422-6.

[3] Sovová Ž, Štikarová J, Kaufmanová J, Májek P, Suttnar J, Šácha P et al. Impact of posttranslational modifications on atomistic structure of fibrinogen. PloS one 2020;15:e0227543.

[4] Abraham MJ, Murtola T, Schulz R, Páll S, Smith JC, Hess B et al. GROMACS: High performance molecular simulations through multi-level parallelism from laptops to supercomputers. SoftwareX 2015;1:19-25.

[5] Schmid N, Eichenberger AP, Choutko A, Riniker S, Winger M, Mark AE et al. Definition and testing of the GROMOS force-field versions 54A7 and 54B7. European biophysics journal 2011;40:843.

[6] Petrov D, Margreitter C, Grandits M, Oostenbrink C, Zagrovic B. A systematic framework for molecular dynamics simulations of protein post-translational modifications. PLoS computational biology 2013;9:e1003154.

[7] Berendsen HJ, Postma JP, van Gunsteren WF, Hermans J. Interaction models for water in relation to protein hydration. In: Anonymous Intermolecular forces: Springer; 1981, p. 331-342.

[8] Bussi G, Donadio D, Parrinello M. Canonical sampling through velocity rescaling. J Chem Phys 2007;126:014101.

[9] Berendsen HJ, Postma Jv, van Gunsteren WF, DiNola A, Haak JR. Molecular dynamics with coupling to an external bath. J Chem Phys 1984;81:3684-90.

[10] Tironi IG, Sperb R, Smith PE, van Gunsteren WF. A generalized reaction field method for molecular dynamics simulations. J Chem Phys 1995;102:5451-9.

[11] Hess B, Bekker H, Berendsen HJ, Fraaije JG. LINCS: a linear constraint solver for molecular simulations. Journal of computational chemistry 1997;18:1463-72.
